# The impact of analytical treatment interruptions and trial interventions on time to viral re‐suppression in people living with HIV restarting ART in cure‐related clinical studies: a systematic review and meta‐analysis

**DOI:** 10.1002/jia2.26349

**Published:** 2024-08-18

**Authors:** Ming Jie Lee, Miles Eason, Antonella Castagna, Galli Laura, Marie‐Angelique De Scheerder, James Riley, Pablo Tebas, Jesper Gunst, Ole Søgaard, Eric Florence, Eugene Kroon, Mark De Souza, Beatriz Mothe, Marina Caskey, Sarah Fidler

**Affiliations:** ^1^ Department of Infectious Disease Imperial College London London UK; ^2^ Peter Medawar Building for Pathogen Research, Nuffield Department of Medicine University of Oxford Oxford United Kingdom; ^3^ Faculty of Medicine Imperial College London London UK; ^4^ Clinic of Infectious Diseases Vita‐Salute University San Raffaele Scientific Institute Milan Italy; ^5^ Infectious Diseases Istituto di Ricovero e Cura a Carattere Scientifico (IRCCS) San Raffaele Scientific Institute Milan Italy; ^6^ Department of General Internal Medicine Ghent University Hospital Gent Belgium; ^7^ Center for Cellular Immunotherapies Department of Microbiology Perelman School of Medicine University of Pennsylvania Philadelphia Pennsylvania USA; ^8^ Department of Infectious Diseases Aarhus University Hospital Aarhus Denmark; ^9^ Department of Clinical Medicine Aarhus University Aarhus Denmark; ^10^ Departments of Clinical and Biomedical Sciences Institute of Tropical Medicine Antwerp Belgium; ^11^ Department of Infectious Diseases University Hospital Antwerp Belgium; ^12^ SEARCH Research Foundation Bangkok Thailand; ^13^ IrsiCaixa AIDS Research Institute Hospital Germans Trias i Pujol Badalona Spain; ^14^ Laboratory of Molecular Immunology The Rockefeller University New York New York USA

**Keywords:** antiretroviral therapy, ATI, HIV, HIV cure, treatment interruption, viral suppression

## Abstract

**Introduction:**

To assess the effectiveness of novel HIV curative strategies, “cure” trials require periods of closely monitored antiretroviral therapy (ART) analytical treatment interruptions (ATIs). We performed a systematic review and meta‐analysis to identify the impact of ATI with or without novel therapeutics in cure‐related studies on the time to viral re‐suppression following ART restart.

**Methods:**

Medline, Embase and Web of Science databases were searched for human studies involving ATIs from 1 January 2015 till 22 April 2024. The primary outcome was time to first viral re‐suppression (plasma HIV viral load [VL] <50 copies/ml) stratified by receipt of interventional drug with ATI (IA) or ATI‐only groups. Random‐effects proportional meta‐analysis and multivariable Cox proportional hazards analysis were performed using R.

**Results:**

Of 1073 studies screened, 13 were included that met the inclusion criteria with VL data available after restarting ART (*n* = 213 participants). There was no difference between time to viral suppression in IA or ATI‐only cohorts (*p* = 0.22). For 87% of participants, viral suppression within 12 weeks of ART restart was achieved, and all eventually had at least one VL <50 copies/ml during follow‐up. After adjusting for covariables, while participants in the IA cohort were associated with less rapid suppression (adjusted hazard ratio [aHR] 0.61, 95% CI 0.40–0.94, *p* = 0.026), other factors include greater log VL at ART restart (aHR 0.56, 95% CI 0.46–0.68, *p*<0.001), duration since HIV diagnosis (aHR 0.93, 95% CI 0.89–0.96) and longer intervals between HIV VL monitoring (aHR 0.66, 95% CI 0.59–0.74, *p*<0.001). However, the use of integrase inhibitors was associated with more rapid viral suppression (aHR 1.74, 95% CI 1.16–2.59).

**Discussion:**

When designing studies involving ATIs, information on time to viral re‐suppression after restarting ART is important to share with participants, and should be regularly monitored and reported, to assess the impact and safety of specific trial interventions in ATI studies.

**Conclusions:**

The majority of participants achieved viral suppression after restarting ART in ATI studies. ART regimens containing integrase inhibitors and frequent VL monitoring should be offered for people restarting ART after ATI studies to ensure rapid re‐suppression.

## 1 INTRODUCTION

Antiretroviral therapy (ART) has dramatically improved survival for the 40 million people living with HIV globally; however, ART alone cannot cure HIV infection. Drawbacks to current ART still include adherence requirements, uncertain long‐term safety profiles, treatment fatigue, drug resistance, cost and challenges to sustained delivery through healthcare systems [1]. Therefore, the search for safe, effective longer‐acting treatment, or long‐term remission strategies for HIV remains a priority.

Without a validated marker available for predicting the effectiveness of HIV remission and cure strategies, analytical treatment interruption (ATI) of ART remains the only method to do so. ATIs are defined as closely monitored and usually temporary interruptions of ART during a study to assess markers of immunological and viral reservoir control. While recommendations are available for conducting studies involving ATIs [2], there is often considerable heterogeneity in the criteria used to re‐start ART; however, recent studies involving ATIs were often of shorter duration, more intensely monitored and had more conservative viral load (VL) thresholds to restart ART [3]. A systematic review and meta‐analysis reported that studies with more frequent VL monitoring, high baseline CD4 counts and undetectable VLs were safe with negligible risk of adverse events (0%, 95% CI 0–1%) or development of ART drug resistance (0%, 95% CI 0–1%) [4].

Although safety data are routinely available during the period of ATI in clinical trials, there is often limited data or recommendations on viral re‐suppression rates following ART reinitiation after ATI. Studies have reported that the reservoir containing total and replication‐competent HIV remains stable following short periods of ATI [5, 6, 7], but the small sample sizes and short duration of ATI may limit the generalizability of their findings to larger studies with longer periods of treatment interruption. It also remains unclear what effect novel immunotherapeutics such as broadly neutralizing antibodies (bNAbs) have on the HIV reservoir following ATI [6], one study suggests that bNAbs were associated with a modest reduction in the intact proviral reservoir [8]. For participants living with HIV and their healthcare providers, the concerns of onward HIV transmission remain the greatest concern when considering participating in clinical trials involving ATIs [9], and data to demonstrate the safety and ability to achieve viral suppression after restarting ART in ATI studies are paramount to addressing these concerns.

In this systematic review and meta‐analysis, we analysed recent studies to determine the proportion of people living with HIV participating in ATI cure‐related clinical studies, who achieve viral suppression following ART re‐initiation. We assessed the impact of receiving any trial interventions on viral re‐suppression rates compared to undertaking ATI without interventions (ATI only), as well as other participant characteristics and trial‐design factors associated with the time to viral re‐suppression.

## 2 METHODS

### 2.1 Search strategy and selection process

A structured systematic literature search of the Medline, Embase and Web of Science databases was performed, an initial search on 2 March 2023, and an updated literature search on 22 April 2024. Two reviewers (ME and MJL) read and ascertained the relevance of the studies independently. Discordant results were adjudicated by a third reviewer (SF). The full protocol was registered on Prospero registry (Registration Number CRD42023403809) and followed the recommendations from the PRISMA statement (Table S1). Full search terms and results from the final literature search are available in Tables S2–S5.

#### 2.1.1 Inclusion criteria

Clinical studies with an ATI were included. An ATI was defined as a protocol‐determined temporary pause in ART with a viral rebound and regular VL measurements. Only studies with viral suppression data after ART restart were included, defined as studies with plasma VL measurements for participants until at least one VL <50 copies/ml. Frequency of VL monitoring, originally defined as at least fortnightly in the original inclusion criteria on the Prospero registry, was dropped to increase the number of studies available and allow analysis of the association of frequency of VL monitoring on the time to VL suppression and all studies with at least one VL measurement post‐ART were included.

#### 2.1.2 Exclusion criteria

Any studies prior to 2015, pre‐prints, abstracts, letters, reviews, commentary articles, opinion articles, animal studies and in vitro studies were excluded. This timeframe was selected to include studies that included integrase inhibitors. Studies with children and adolescent participants (<18 years old) were excluded, and stem cell transplant recipients were also excluded due to the unique characteristics of participants and distinct mechanism of action featuring potentially fatal toxicities, their inclusion potentially impacting on the generalizability of the study findings. Studies where VL measurements were not available after restarting ART were not included.

### 2.2 Primary outcome

The time taken to viral suppression <50 copies/ml following ART restart after ATI, stratified by cohorts receiving intervention and undertaking ATI, or undertaking ATI only.

### 2.3 Secondary outcomes

Proportion of participants who experienced viral suppression <50 copies/ml by week 12 after ART restart. Other factors associated with time to viral suppression were analysed using both a univariable and multivariable Cox proportional hazards regression model. Covariables include plasma HIV VL at ART restart, nadir CD4 count, CD4 count at study enrolment, duration since HIV diagnosis, ART regimens, duration of ATI and frequency of HIV VL monitoring after restarting ART.

### 2.4 Data extraction

Extracted data included VL measurements during ATI and after ART restart, participant demographics, HIV‐associated disease characteristics (nadir and baseline CD4 counts, duration since HIV diagnosis, virus clade, presence of ART resistance‐associated mutations), ART regimens restarted, ART restart criteria, interventions received during study and any adverse events including development of drug resistance during and after ATI.

### 2.5 Data collection process and synthesis strategy

ME extracted the data and MJL was responsible for verification of the extracted data. If data were not available from published manuscripts or associated supplementary material, these data were requested from the corresponding study author.

### 2.6 Assessment of quality and bias

As viral re‐suppression rates were not included in the primary or secondary outcomes of these studies included, they were unlikely to drive publication bias on the basis of whether participants achieve viral re‐suppression after restarting ART. Thus, a formal publication bias assessment was not performed beyond a funnel plot assessment presented in Figure S2.

The modified Newcastle Ottawa scale [10] and Cochrane Risk of Bias 2 (ROB2) tool [11] were used to assess the quality and bias of non‐randomized studies and randomized controlled trials (RCTs), respectively. The Grading of Recommendation, Assessment, Development and Evaluation [12] classification was used to evaluate the overall quality of evidence. Two reviewers (ME and MJL) independently assessed bias within each study. Differences were adjudicated by a third reviewer (SF).

### 2.7 Statistical analysis

All analyses were performed using (R version 4.2.2 (2022‐10‐31)) [13]. A Kaplan−Meier curve was plotted stratified by IA or ATI‐alone subgroups to display time‐to‐viral‐suppression results of all study participants. Multivariable time‐to‐event analysis was undertaken using the Cox proportional‐hazards model to examine the effects of different demographics, HIV‐related and study‐related characteristics on the hazard risk (HR) of achieving viral suppression over time. A forward stepwise model approach for variable selection was performed, and variables were included in the multivariable model if there was a univariable association below the threshold of *p* = 0.3. The variables included in the univariate and the final multivariate models are presented in Table 3.

A random effects meta‐analysis was performed using the R package “meta” [13, 14], and the Higgins I^2^ test was used to calculate study heterogeneity. Participants from studies with multiple arms were pooled within cohorts undertaking IA or ATI‐only protocols and treated as separate cohorts for the proportional meta‐analysis. A stratified meta‐analysis of proportion effect estimates (ES) compared participants in the IA subgroup with participants in the ATI‐alone subgroups, and results were presented in forest plots and pooled ES with 95% confidence intervals, using χ^2^‐squared test to assess for subgroup differences.

A sensitivity analysis was performed to assess the weight of the effect of bNAbs alone compared to all interventions received. Participants who received any other interventional therapies apart from bNAbs were excluded, and the Cox proportional‐hazards model was repeated.

## 3 RESULTS

**Figure 1:**
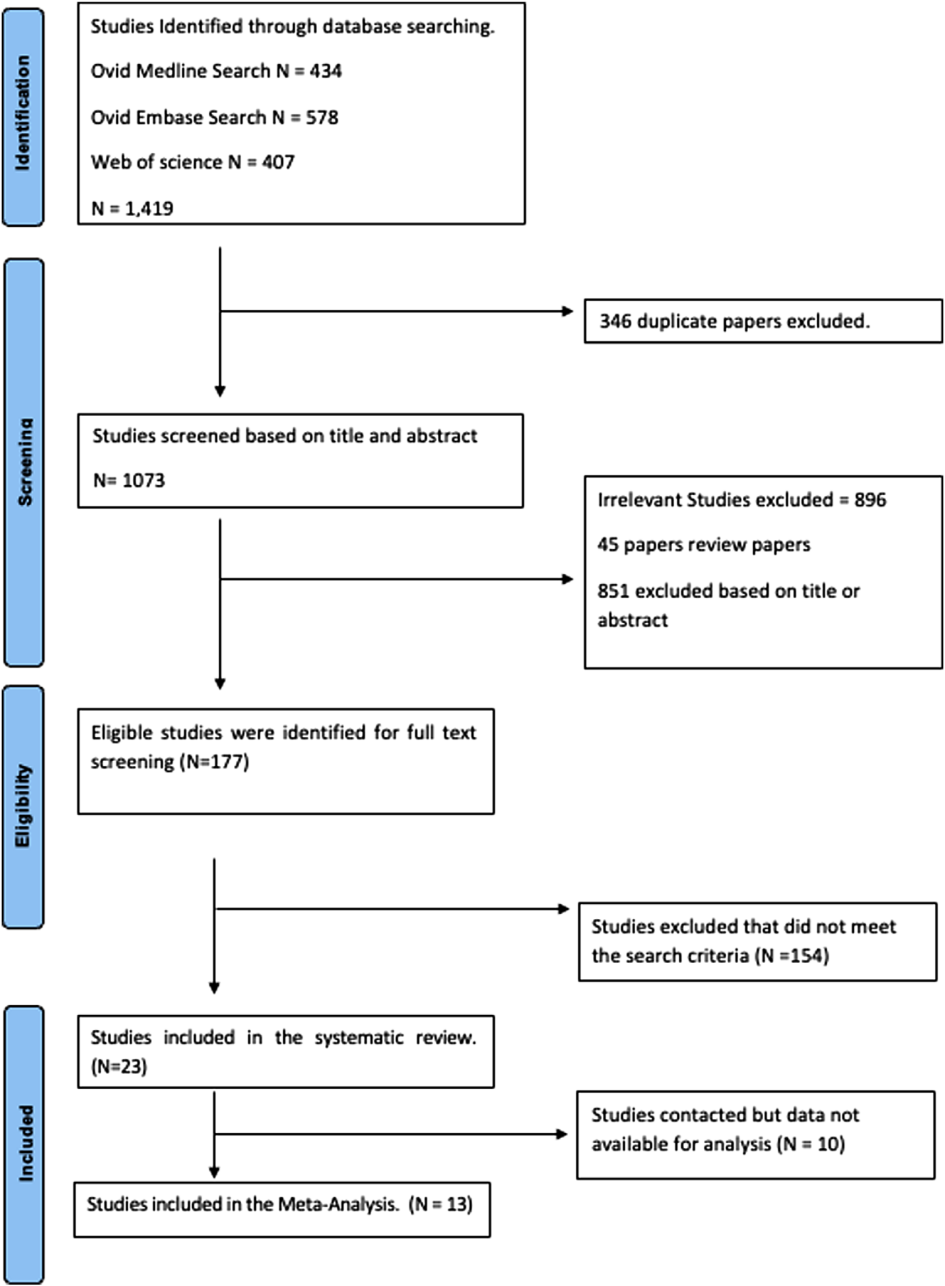
Flow chart of the study selection process.

Of 1073 papers screened under the search terms, the initial search resulted in 22 studies involving ATIs in their study design which met all inclusion and exclusion criteria [8, 15, 16, 17, 18, 19, 20, 21, 22, 23, 24, 25, 26, 27, 28, 29, 30, 31, 32, 33, 34, 35], and the updated literature search revealed a further study [36], resulting in 23 studies in total. Of these studies, 13 studies published or communicated sufficient data for inclusion in this systematic review (Figure 1). Authors from the remaining 10 studies were contacted but were unable to provide the necessary VL data following ART restart within the timeframe requested, and these studies were excluded from the meta‐analysis as per the exclusion criteria. Characteristics and study design of the 23 studies are summarized in Table 1.

**Table 1:** Summary of studies

First author	Publication year and journal	Number of participants who underwent ATI	Intervention with ATI or ATI only	Details of study	VL criteria to restart ART	Monitoring frequency of VL after ART restart	Sex	Median age (years)	Median ART duration prior to study entry (years)	Median CD4^+^ count at baseline (cells/µl)	Post‐ART restart data status
Castagna	J Antimicrob Chemother 2019	9	ATI only	Observational ATI study	Two consecutive HIV VL >50 copies/ml (14 days apart)	Not stated in manuscript	9 M	50.7	16.9	737	Available included in the meta‐analysis
Mendoza	Nature 2018	15	Intervention with ATI	Open‐label single‐arm. Participants included in this study received three infusions of 30 mg/kg 10–1074 + 3BNC117 antibodies. They then had an ATI.	Two consecutive measurements of HIV VL ≥200 copies/ml	Fortnightly until <50 copies/ml. Every 2 weeks thereafter.	1 F 14 M	40	5	654	Available included in the meta‐analysis
Scheid	Nature 2016	13	Intervention with ATI	Non‐randomized dose finding study **Arm 1**: Two doses of 3BNC117 + ATI **Arm 2**: Four doses of 3BNC117 + ATI	Two consecutive measurements of HIV VL ≥200 copies/ml (weekly measurements)	Weekly	1 F 12 M	31	6	720	Available included in the meta‐analysis
Gaebler	Nature 2022	26	Intervention with ATI	Open‐labelled RCT. **Arm 1**: Received bNAbs 3BNC117 and 10–1074 during ATI. **Arm 2**: Took ART while receiving 3BNC117 and 10–1074 and then underwent ATI.	**Weeks 0–26**: Two consecutive measurements of HIV VL ≥200 copies/ml (weekly measurements) **Weeks 26–38**: Four weekly HIV VL >1000 copies/ml; **Weeks 38–48**: Two consecutive pVL >1000 measured every other week	Monthly	3 F 23 M	49.5	10	675	Available included in meta‐analysis
Gruell	Lancet Microbe 2022	20	Intervention with ATI	**Group 1** underwent two treatment cycles of antibody each consisting of 3BNC117 infusions (30 mg/kg) + three romidepsin infusions (5 mg/m^2^). **Group 2** Two treatment cycles each consisting of three romidepsin infusions (5 mg/m^2^).	Two consecutive measurements of HIV VL ≥200 copies/ml	Fortnightly until <20 copies/ml	3 F 17 M	Arm 1: 40 Arm 2: 51	Arm 1: 5 years Arm 2: 10 years	Arm 1: 716 Arm 2: 600	Available included in the meta‐analysis
Cohen	J Exp Med 2018	15	Intervention with ATI	Open label. Received 3BNC117 infusions and then had an ATI.	Two consecutive measurements of HIV VL ≥200 copies/ml	Weekly	1 F 14 M	43	11	688	Available included in the meta‐analysis
Gunst	Nat Med 2022	20	Intervention with ATI	Open‐labelled RCT. **Arm 1**: ATI only **Arm 2**: ART+3BNC117 then ATI **Arm 3**: ART + romidepsin then ATI **Arm 4**: ART + 3BNC117 + romidepsin then ATI	Two consecutive measurements of HIV VL ≥5000 copies/ml (weekly measurements)	Every fourth week until VL were undetectable on two consecutive measurements.	2 F 18 M	37	0	529	Available included in the meta‐analysis
Tebas	J Clin Invest 2021	14	Intervention with ATI	Open‐labelled study. **Arm 1**: Gene modified CCR5 CD4^+^ T cells. **Arm 2**: Gene modified CCR5 CD4^+^ T cells + CTX and then ATI.	Consecutive measurements of HIV VL ≥100,000 copies/ml over 3 weeks	Monthly	1 F 13 M	44	N/A	693	Available included in the meta‐analysis
Kroon	J Virus Erad 2020	15	Intervention with ATI	Open‐labelled RCT. **Arm 1**: Vorinostat/Hydroxychloroquine/Maraviroc + ART followed by ATI **Arm 2**: ART followed by ATI only.	Two consecutive measurements of HIV VL ≥200 copies/ml	Fortnightly until 34 weeks after	2 F 13 M	Group 1: 28 Group 2: 26	3.4	Group 1: 634 Group 2: 1079	Available included in the meta‐analysis
De Scheerder	Cell Host Microbe 2022	11	ATI only	Observational ATI study	Two consecutive measurements of HIV VL ≥200 copies/ml (3 days apart) or a single HIV VL ≥1000 copies/ml	Post‐ART restart at 4 weeks and at 12 weeks. If detectable at 4 weeks, VL repeated monthly until undetectable.	1 F 10 M	40	3.5	688	Available included in the meta‐analysis
Pannus	J Int AIDS Soc 2020	16	ATI only	Observational ATI study	Two consecutive measurements of HIV VL ≥1000 copies/ml (3 days apart) or a single HIV VL ≥10,000 copies/ml	Post‐ART restart at 4 weeks and at 12 weeks.	15 M 1 F	N/A	4	N/A	Available included in the meta‐analysis
Bailón	Nat Med 2022	45	Intervention with ATI	Double‐blinded RCT. **Arm 1**: Received a combination of DNA.HTI, MVA.HTI and ChAdOx1.HTI l vaccines and then an ATI. **Arm 2** Received ATI + placebo	Eight consecutive measurements of HIV VL ≥10,000 copies/ml or a single HIV VL ≥100,000 copies/ml	Post‐ART restart HIV VL was monitored at weeks 4 and 12.	44 M 1 F	36	N/A	745	Available included in the meta‐analysis
Gunst	Nat Med 2023	46	Intervention with ATI	Double‐blinded RCT Arm 1: Placebo/placebo (P/P) Arm 2: Lefitolimiod/placebo (L/P) Arm 3: Placebo/bNAb (P/B) Arm 4: Lefitolimod/bNAb (L/B)	Sustained HIV VL ≥1000 copies/ml for ≥4 weeks or confirmed VL ≥ 100,000 copies/ml	Monthly	39 M 7 F	Age by arm: P/P 54 L/P 44 P/B 51 L/B 48	Time on ART by arm (years): P/P 8 L/P 9 P/B 8 L/B 8	CD4 count by arm P/P 743 L/P 694 P/B 1027 L/B 832	Available for 33 of 46 participants, included in the meta‐analysis
Leal	Front Immunol 2021	29	Intervention with ATI	RCT. Testing Intranodal vaccine. **Arm 1**: DCV3 **Arm 2**: DCV3 with PEG‐INF **Arm 3**: Placebo **Arm 4**: Placebo + PEG‐INF	A single HIV VL ≥100,000 copies/ml	Every 4 weeks until 12 weeks after ART restart.	29 M	46	N/A	752	Not available. Not included in meta‐analysis.
Bar	NEJM 2016	24	Intervention with ATI	Combination of two open‐label studies (ACTG 5340 + NIH trial 15‐I‐0140) of VCR01.	A sustained (≥2 weeks) HIV VL >1000 copies/ml; HIV VL ≥200 copies/ml followed by a confirmation level of 1000 copies/ml or three consecutive measurements of >200 copies/ml	Monthly	22 M 2 F	A530 cohort 38 NIH trial 51	A530 cohort 4.7 NIH trial 10.0	A530 cohort 896 NIH trial 724	Not available. Not included in meta‐analysis
Sneller	J Infect Dis 2020	22	ATI only	Observational ATI study	HIV VL >1000 copies/ml for ≥ 4 weeks	HIV VL measured weekly for the first 4 weeks then monthly thereafter for up to 52 weeks	20 M 2 F	51	7.7	767	Not available Not included in the meta‐analysis
Crowell	Lancet HIV 2019	18	Intervention with ATI	Double‐blinded RCT. Participants either received VRC01 or a placebo and ATI.	Confirmed HIV VL >1000 copies/ml	Not stated in manuscript	18 M	29	3	717	Not available. Not included in the meta‐analysis.
Colby	Nat Med 2020	26	Intervention with ATI	RCT. Receive vaccine or placebo.	Two consecutive measurements of HIV VL ≥200 copies/ml (weekly measurements)	Not stated in manuscript	26 M	25	1.9	625	Not available. Not included in the meta‐analysis.
SenGupta	Science Transl Med 2021	25	Intervention with ATI	RCT. Participants received either vestolimod or placebo and then had ATI.	Not stated in manuscript	Not stated in manuscript	21 M 4 F	52 Vestilamod 41 Placebo	2.7 Vestilamod 3.2 Placebo	752 Vestilamod 952 Placebo	Not available. Not included in the meta‐analysis.
Liu	J Clin Invest 2021	6	Intervention with ATI	Open‐labelled CAR T cells study	Two consecutive measurements of HIV VL ≥200 copies/ml	Every 3 weeks	6 M	30	3.9	530	Not available. Not included in the meta‐analysis.
Rutsaert	J Virus Erad 2019	30	Intervention with ATI	RCT **Arm 1**: 50 mg ABX464 and ATI **Arm 2**: 150 mg ABX464 and ATI **Arm 3**: Placebo and ATI	Not available in the manuscript	Every 14 days until viral suppression	29 M 1 F	Arm 1 45.2 Arm 2 38.4 Arm 3 48.3	N/A	Arm 1: 1004 Arm 2: 926 Arm 3: 698	Not available. Not included in the meta‐analysis.
Vibholm	AIDS 2019	9	Intervention with ATI	**Arm 1**: MGN1703 with ART and then ATI **Arm 2**: ART with MGN1703 and ATI with MGN1703	Two consecutive measurements of HIV VL ≥5000 copies/ml	Not stated in manuscript. Only states they were followed until suppression.	11 M 1 F	51.5	6.3	635	Not available. Not included in the meta‐analysis.
Colby	Nat Med 2018	8	ATI only	Observational ATI study	Confirmed HIV VL >1000 copies/ml HIV VL rise of ≥0.5 log_10_ copies/ml per day provided that the last VL was >1000 copies/ml, a single HIV VL >10,000 copies/ml	Not stated in the manuscript	7 M 1 F	29	2.8	577	Not available. Not included in the meta‐analysis.

### 3.1 Characteristics of the studies included in the meta‐analysis

This meta‐analysis included three observational studies and 10 interventional studies, of which six were RCTs and four were non‐randomized trials. The interventions studied include bNAb in seven studies [8, 16, 17, 18, 19, 20, 36], and individual studies involving HIV vaccines [33], latency reversal agents only [22] and CCR5 gene‐edited T‐cell infusions [21]. The three observational studies were of participants undertaking ATIs only without any intervention [15, 23, 24].

The exact criteria to restart ART differed between studies; however, it was determined by plasma HIV VL in all studies (Table 1), with additional criteria based on CD4 thresholds, safety concerns or participant/investigator preferences. Notably, two studies had a much higher VL threshold (>100,000 copies/ml over 1 and 3 weeks, respectively) for ART restart than the other included studies [21, 36]. After the resumption of ART, six studies monitored the VL either weekly or fortnightly, two studies monitored participants’ VL monthly, and two measured VL at weeks 4 and 12 post‐ART restart. There were two studies which did not state in their manuscripts how often VL would be monitored after ART was resumed [15, 20].

### 3.2 Study participant demographics

A total of 213 participants underwent ATI from the 13 included studies in this meta‐analysis. The median age at enrolment was 41 years (range 19−68) and of those studies with sex demographics available, 182 (91.5%) were male, 17 (8.5%) female and 1 (0.5%) transgender female. Median time from HIV diagnosis to enrolment was 5.5 years (range 0–37). Median CD4 count at study enrolment was 706 cells/µl (range 320−2156). Characteristics of study participants undergoing ATI by studies are presented in Table 1.

There were 27/213 (12.7%) participants who did not experience an undetectable VL by week 12 after restarting ART. Table 2 summarizes the characteristics of participants who did not experience viral suppression by week 12. Of these participants, 20 had received an interventional study drug (eight bNAbs, three bNAbs with TLR‐9 agonist, four TLR‐9 agonist, five CCR5‐edited CD4 T cells with or without cyclophosphamide and one who received romidepsin only) and seven underwent ATI‐only protocols. They were almost all male (26/27), and the median age was 51 years old (range 31–62). Due to the frequency of VL monitoring after restarting ART, it is possible that nine participants may have achieved undetectable VL results within 12 weeks of restarting ART if monitored more frequently. Of the remaining 19 participants with detectable VL results >50 copies/ml beyond 12 weeks, they often had higher plasma VL at the time of ART restart (median 74,900 copies/ml, range 12,251–5,610,000 copies/ml), and 7/19 restarted on ART regimens not containing integrase inhibitors (Table 2). There was one individual (807) who did not demonstrate persistent viral re‐suppression and experienced ongoing low‐level viraemia (VL range 44–710 copies/ml) beyond 12 weeks, with only one VL result <50 copies/ml at day 471 after restarting ART.

**Table 2:** Individual characteristics of participants who did not suppress by week 12

Study	Participant ID	ATI with intervention or ATI only	Intervention received	Age	Sex	Clade	Nadir CD4 (cells/µl)	CD4 count at study baseline (cells/µl)	Duration of ATI (weeks)	ART regimen restarted	Mean interval between visits post‐ART restart (weeks)	Viral load at ART restart (copies/ml)	Time taken from ART restart to first undetectable VL (weeks)
Castagna et al. J Antimicrob Chemother 2019	3371	ATI only	ATI only	48	M	F	360	814	3	ABC/3TC/ATV/r	4.2	180,634	12.6
	4844	ATI only	ATI only	51	M	F	402	1512	3	ATV/r	12	12,251	24.0
	5220	ATI only	ATI only	62	M	F	212	586	5	TDF/FTC/EFV	4.7	209,265	28.0
	6127	ATI only	ATI only	50	M	B	386	552	5	TDF/FTC/RPV	6.1	50,396	18.2
Gaebler et al. Nature 2022	5101	Intervention + ATI	3BNC117 + 10–1074	52	M	B	Not available	671	8	TDF/FTC/DTG	3.3	639,760	20.0
	5125	Intervention + ATI	3BNC117 + 10–1074	34	M	B	450	1006	33	TDF/FTC/DTG	3	19,300	15.0
Gruell et al. Lancet Microb 2022	03‐16‐B	Intervention + ATI	Romidepsin	62	M	B	Not available	Not available	6	TAF/FTC/EVG/c	4.7	57,070	14.0
Mendoza et al. Nature 2018	9248	Intervention + ATI	3BNC117 + 10–1074	52	M	B	310	620	13	TAF/FTC/DRV/r	1.9	18,200	13.0
	9249	Intervention + ATI	3BNC117 + 10–1074	49	M	B	426	1070	4	ABC/3TC/DRV/r	2.2	60,000	20.0
Tebas et al. J Clin Invest 2021	101	Intervention + ATI	CCR5‐edited CD4^+^ T cells	32	M	Not available	Not available	563	16	TDF/FTC/EFV	4.275	9637	17.1
	102	Intervention + ATI	CCR5‐edited CD4^+^ T cells	49	M	Not available	Not available	870	16	TDF/FTC/ATV/r	4	28,435	16.0
	202	Intervention + ATI	CCR5‐edited CD4^+^ T cells + CTX	60	M	Not available	Not available	512	17	TDF/FTC/RPV	4.5	56,198	18.0
	205	Intervention + ATI	CCR5‐edited CD4^+^ T cells + CTX	45	M	Not available	Not available	456	16	TDF/FTC/ATV/r	5.3	35,910	15.8
De Scheerder et al. Cell Host Microbe 2019	STAR 002	ATI only	ATI only	38	M	B	438	2000	8	TAF/FTC/EVG/c	8.6	59,900	25.7
Gunst et al. Nat Med 2023	109	Intervention + ATI	3BNC117 + 10–1074	57	M	B	Not available	1410	10	ABC/3TC/DTG	Not available (monthly in protocol)	43,600	14.9
	114	Intervention + ATI	Lefitolimod + 3BNC117 + 10–1074	31	M	B	Not available	610	26	TDF/FTC/DRV/c		40,100	18.4
	115	Intervention + ATI	Leftolimod	53	M	Not available	Not available	840	4	TAF/FTC/DTG		1,150,000	31
	119	Intervention + ATI	3BNC117 + 10–1074	50	M	B	Not available	780	12	TAF/FTC/BIC		74,900	18.1
	134	Intervention + ATI	Leftolimod	58	M	B	Not available	600	6	TAF/FTC/BIC		32,200	17.1
	601	Intervention + ATI	3BNC117 + 10–1074	52	M	B	Not available	Not available	30	TDF/FTC/DTG		173,000	17.0
	801	Intervention + ATI	Leftolimod + 3BNC117 + 10–1074	52	M	B	Not available	1076	11	ABC/3TC/DTG		2,100,000	18.4
	822	Intervention + ATI	Leftolimod + 3BNC117 + 10–1074	54	M	B	Not available	703	8	ABC/3TC/DTG		400,000	15.3
	301‐13	Intervention + ATI	Leftolimod	31	F	C	Not available	750	6	ABC/3TC/DTG		328,000	15
	301‐4	Intervention + ATI	Leftolimod	44	M	Not available	Not available	630	3	TDF/FTC/DTG		5,610,000	23.0
	807^a^	Intervention + ATI	3BNC117 + 10–1074	40	M	C	Not available	1561	14	ABC/3TC/DTG		370,000	67.3
	205	ATI only	ATI only	54	M	B	Not available	Not available	12	TAF/FTC/DRV		18,000	13.3
	502	ATI only	ATI only	61	M	B	Not available	930	5	ABC/3TC/DRV/r		333,000	20.1

^a^ This participant continued to experience low‐level viraemia during the follow‐up period 2 years after restarting ART.

There were no adverse events reported in the included studies related to restarting ART after the ATI.

### 3.3 Multivariable time‐to‐event analysis

**Figure 2:**
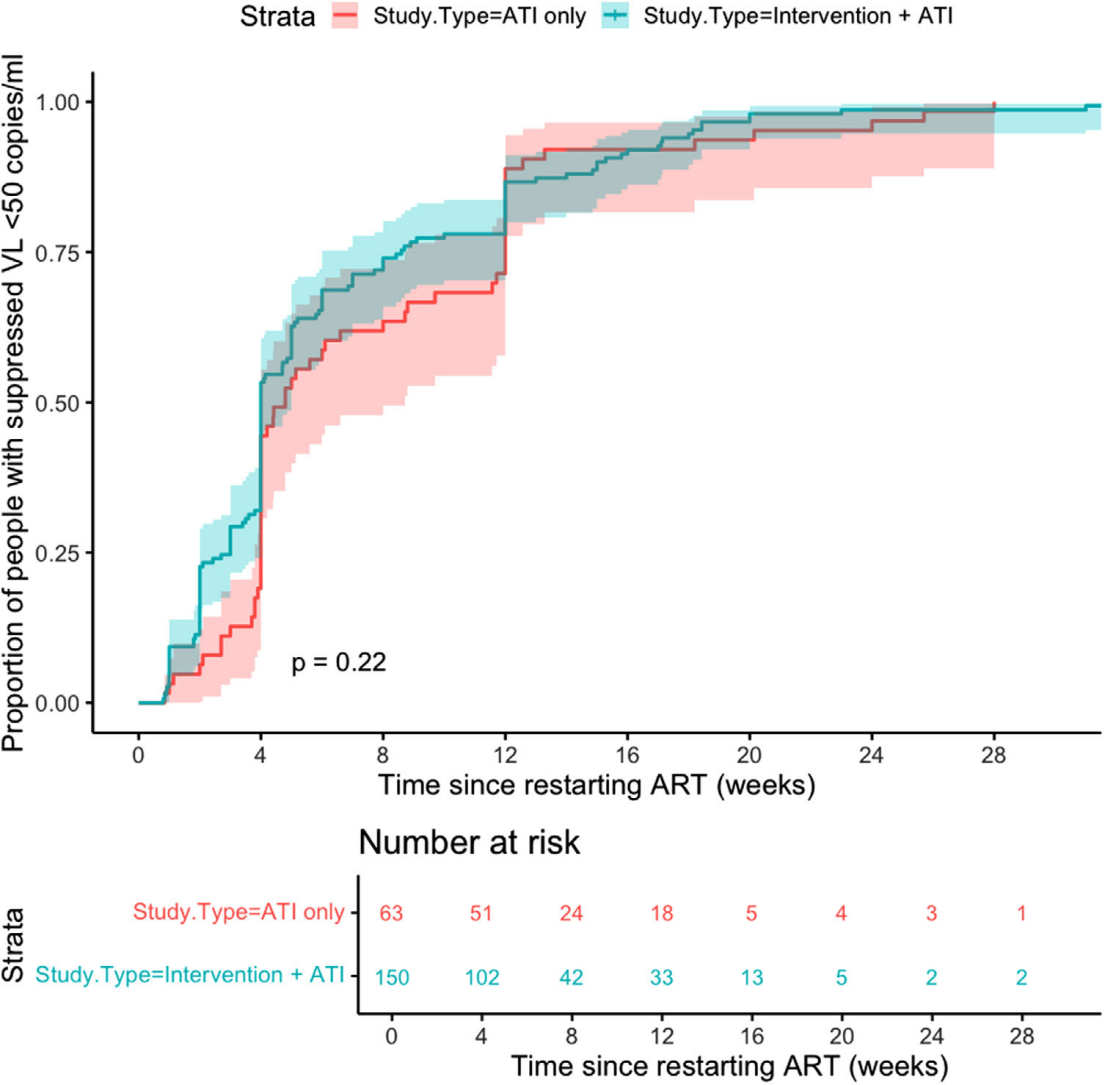
Kaplan−Meier plot of the proportion of participants with undetectable plasma viral loads (<50 copies/ml) following ART restart after ATI (weeks). Kaplan−Meier survival plot of the proportion of participants with suppressed plasma viral load <50 copies/ml against time in weeks. The red line represents participants undergoing ATI‐only protocols, and the blue line represents participants who received an interventional study drug with ATI as part of their study protocol. The red and blue shaded areas represent the 95% confidence intervals of their respective lines. Large changes in proportions at weeks 4 and 12 reflect study protocols where monitoring was undertaken only at these times after restarting ART. The table below shows the numbers at risk at 4‐weekly intervals stratified by receipt of interventional study drug with ATI or ATI‐only protocols. Datapoints are censored past week 32. Abbreviations: ART, antiretroviral therapy; ATI, analytical treatment interruption; VL, viral load.

There was no difference in time to viral suppression between participants in the IA group compared to those who underwent ATI alone (*p* = 0.22) (Figure 2). When additional variables were adjusted for in a Cox proportional‐hazards model (Table 3), participants in the IA group were less likely to achieve viral suppression compared to those undergoing ATI alone (adjusted hazard ratio [aHR] 0.61, 95% CI 0.40–0.94, *p* = 0.026). A greater plasma HIV VL at ART restart (log copies/ml) (aHR 0.47, 95% CI 0.38–0.59, *p*<0.001), a longer interval between VL measurements after restarting ART (aHR 0.60, 95% CI 0.52–0.68, *p*<0.001) were associated with less likelihood of achieving viral re‐suppression over time, and the use of integrase inhibitors containing regimens was associated with greater likelihood of achieving viral suppression over time (aHR 1.88, 95% CI 1.25–2.82, *p* = 0.002). There was also a small effect associated with the duration since HIV diagnosis (aHR 0.93, 95% CI 0.90–0.97, *p* <0.001). All participants in the included studies eventually achieved at least one VL reading <50 copies/ml after restarting ART (range 12.6−67.3 weeks).

**Table 3:** Multivariable Cox proportional‐hazards regression model

		Univariable			Multivariable		
	*N* (%)/Mean (SD)	HR	95% CI	*p*‐value	HR	95% CI	*p*‐value
**Patient characteristics**							
Age (years) (per 10‐year increase)	41.7 (10.7)	0.80	0.70–0.91	0.001	0.97	0.81–1.15	0.719
Sex^a^							
Female (Ref)	17 (8.5%)						
Male	182 (91.5%)	0.70	0.43–1.16	0.171	0.72	0.40–1.31	0.282
**HIV‐related characteristics**							
Plasma HIV viral load at ART restart (log copies/ml)	4.4 (0.9)	0.56	0.48–0.66	<0.001	0.56	0.46–0.68	<0.001*
Nadir CD4 (per 100 cells/µl increase)	544.6 (287.5)	1.00	0.94−1.06	0.914			
CD4 at study enrolment (per 100 cells/µl increase)	777.3 (299.8)	0.95	0.91−1.00	0.039	0.96	0.91–1.01	0.155
Duration since HIV diagnosis (years)	7.4 (7.4)	0.97	0.95 0.99	0.008	0.93	0.89–0.96	<0.001*
ART regimen containing integrase inhibitors							
No (Ref)	52 (24.5%)						
Yes	161 (75.5%)	1.19	0.86–1.63	0.286	1.74	1.16–2.59	0.007*
**Study characteristics**							
Duration of ATI in weeks	9.9 (8.1)	1.00	0.99 – 1.02	0.830			
Frequency of HIV VL monitoring between ART restart and viral suppression (weeks between visits)	3.4 (2.1)	0.73	0.67–0.79	<0.001	0.59	0.52–0.68	<0.001*
Study type							
ATI only (Ref)	63 (29.6)						
IA	150 (70.4)	1.18	0.88–1.58	0.279	0.61	0.40–0.94	0.026*

*Note*: Significant results are marked by *.

Abbreviations: ART, antiretroviral therapy; ATI, analytical treatment interruption; HR, hazard ratio; IA, intervention with ATI protocol; Ref, reference category.

^a^ A transgender participant was not included in the multivariable analysis due to *n* <5.

In the sensitivity analysis, there was a non‐significant trend towards more rapid time to viral suppression for participants who receive bNAbs without any other interventions compared to those undergoing ATI alone (*p* = 0.055) (Figure S1). However, this trend was reversed once adjusted for covariables (aHR 0.0.28, 95% CI 0.16–0.51, *p* = <0.001). Other covariables associated with time to viral suppression were consistent with the primary outcome analysis. (Table S4).

### 3.4 Meta‐analysis

**Figure 3:**
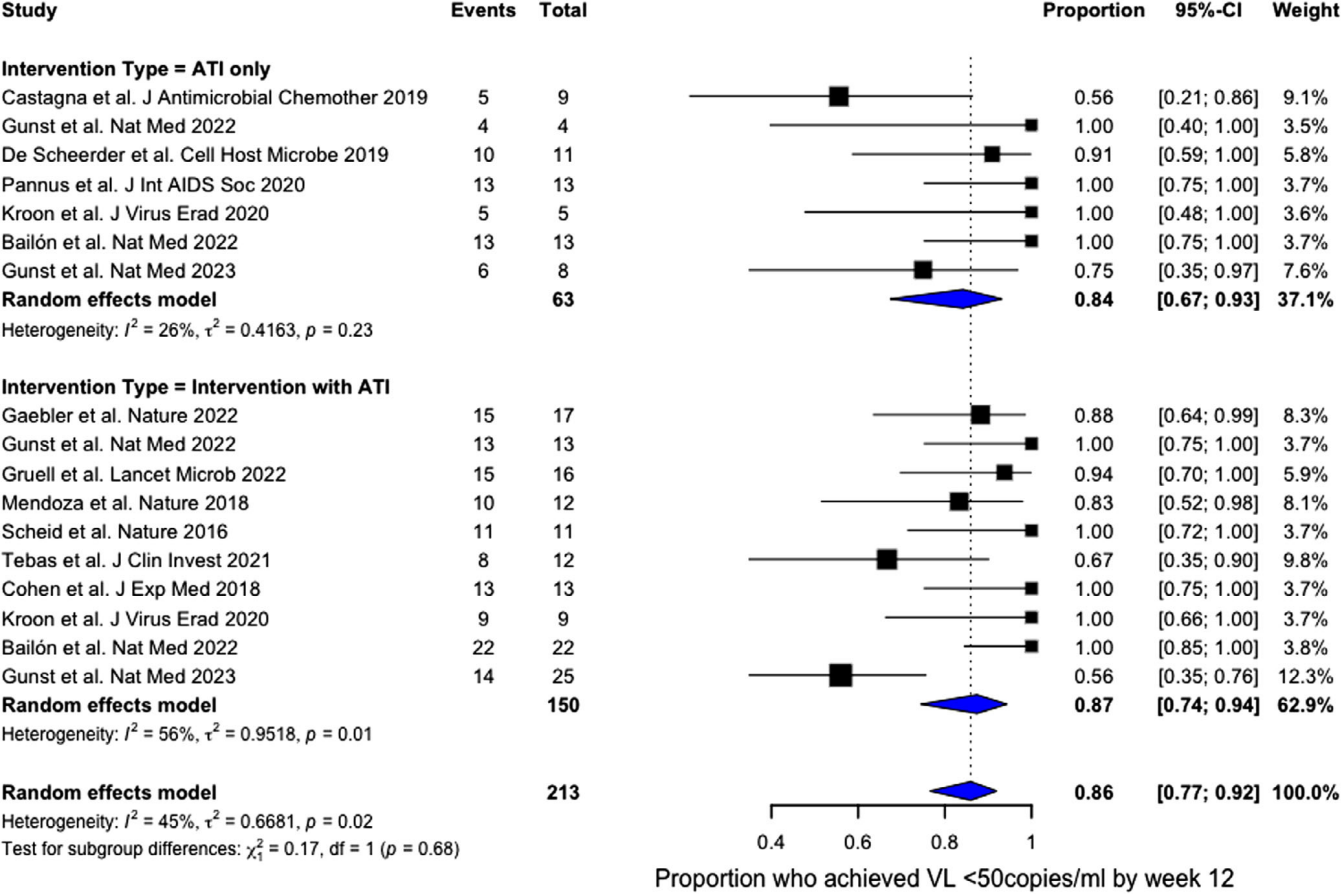
Forest plots of primary outcome events stratified by receipt of intervention with ATI or undergoing ATI‐only protocols. Studies are identified by the name of the first author, journal and year of publication. Primary outcome events were defined as participants who experienced viral suppression with plasma HIV viral load <50 copies/ml) by week 12 after restarting ART. Study weights (%) are from the random‐effects analysis and represented by individual box sizes. The dashed line represents the overall proportion of primary outcome events across all participants. Abbreviations: ATI, analytical treatment interruption; CI, confidence interval.

The pooled proportion of participants who experienced viral suppression <50 copies/ml within 12 weeks of restarting ART after an ATI was 86% (95% CI 77−92%) (Figure 3). There was a moderate degree of heterogeneity present across all studies (I^2^ = 45%, *p* = 0.02). There was no difference between participants who underwent IA compared to ATI‐alone (87% vs. 84%, *p* = 0.68).

### 3.5 Assessment of study quality and bias

The modified Newcastle Ottawa scale [10] was used to assess for bias in the single‐arm cohort and observational studies (Table 4). All studies had a predominantly male population, with strict co‐morbidities exclusion criteria. Three studies with longer follow‐up intervals (monthly or greater) were considered likely to introduce a bias for obtaining the primary outcome (Table 4). Bias was assessed for RCTs using the Cochrane ROB2 tool [11] and four studies assessed raised some concerns for bias raised in domain 3 (missing outcomes data) and domain 4 (measurement of the outcome) due to the limited VL monitoring following ART restart likely contributing to bias in the time to achieving viral suppression, due to the knowledge of intervention arms that may influence the frequency of monitoring following ART restart, respectively (Table 4).

**Table 4:** Quality and risk of bias assessment

Non‐randomized studies							
Modified Newcastle‐Ottawa Score	Representativeness of the cohort	Ascertainment of exposure	Outcome was not present at the start	Assessment of outcome	Adequate follow‐up duration	Loss‐to follow‐up accounted for	Total
Mendoza et al. Nature 2018	0	1	1	1	1	1	5
Scheid et al. Nature 2016	0	1	1	1	1	1	5
Cohen et al. J Exp Med 2018	0	1	1	1	1	1	5
Castagna et al. J Antimicrob Chemother 2019	0	1	1	1	0	1	4
Tebas et al. J Clin Invest 2021	0	1	1	1	0	1	4
De Scheerder et al. Cell Host Microbe 2022	0	1	1	1	1	1	5
Pannus et al. J Int AIDS Soc 2020	0	1	1	1	0	1	4

Randomized studies							
**Risk of Bias 2 score domains**	**1. Randomization process**	**2. Deviations from intended interventions**	**3. Missing outcome data**	**4. Measurement of the outcome**	**5. Selection of the reported result**		**Overall**
Bailón et al. Nat Med 2022	Low risk	Low risk	Some concerns	Some concerns	Low risk		Some concerns
Gaebler et al. Nature 2022	Low risk	Low risk	Some concerns	Some concerns	Low risk		Some concerns
Gruell et al. Lancet Microbe 2022	Low risk	Low risk	Low risk	Low risk	Low risk		Low risk
Gunst et al. Nat Med 2022	Low risk	Low risk	Some concerns	Some concerns	Low risk		Some concerns
Gunst et al. Nat Med 2023	Low risk	Low risk	Some concerns	Some concerns	Low risk		Some concerns
Kroon et al. J Virus Erad 2020	Low risk	Low risk	Low risk	Low risk	Low risk		Low risk

*Note*: Modified Newcastle‐Ottawa Score: Each score represents the number of stars given per category, to a maximum NOS score of 6 for a well‐designed study with minimal or no bias and 0 for a flawed study with substantial bias across all domains. Green cells highlights the domains or overall scores with low risk of bias, and yellow cells highlights domains or overal scores with some concerns of bias.

Although the funnel plot showed asymmetry in the publications (Figure S2), this is unlikely to be due to publication bias as discussed earlier. Alternative explanations may include participants demonstrating late viral suppression associated with high VLs prior to restarting ART, lower rates of integrase inhibitor use and infrequent VL monitoring which led to their outlier positions with low standard error rates.

## 4 DISCUSSION

This is the first systematic review and meta‐analysis to evaluate the impact of ATIs and interventional therapeutics on the time to viral suppression for participants living with HIV enrolled in cure‐related ATI studies. Overall, there was no difference in time to viral suppression after ATI with or without the receipt of interventional drugs. All participants achieved at least one VL <50 copies/ml, and 87% of participants did so by 12 weeks following ART restart. With current evidence indisputably demonstrating zero risk of sexual transmission when the plasma HIV VL is below 200 copies/ml, and near‐zero risk below 1000 copies/ml [37, 38, 39], the findings of this systematic review provide important reassurance for people considering participation in ATI studies and the impact on subsequent viral suppression.

After adjustment for covariables that impact viral suppression, the receipt of interventional therapeutics appeared to be associated with a longer time to viral re‐suppression, which comprised of bNAbs in the majority of studies included. However, this difference appeared to be negated by the use of integrase inhibitors resulting in no overall difference between IA and ATI‐only cohorts. Other factors that were associated with longer time to viral suppressions were identified in this study, including a greater plasma VL at the time of ART restart, supporting the inclusion of integrase inhibitors when restarting ART at high VL for rapid re‐suppression [40], particularly if they were previously taking ART regimens with a lower barrier to ART resistance.

To explain these findings, there may be hypothetical concerns of novel immunotherapeutics that might stimulate clonal expansion of T cells and active replication of their associated HIV reservoir, leading to persistent viraemia. Latency reversal agents such as romidepsin have been shown to induce detectable viraemia in people living with chronic HIV with previously suppressed VLs, through the activation of replication‐competent proviral reservoir [41]. Immune checkpoint inhibitors may also increase HIV‐specific CD8 T cell activity and concurrently activate HIV transcription leading to transient increases in plasma HIV viraemia [42]. A similar vaccine‐like or “vaccinal” effect may be elicited by bNAbs or their immune complexes through the priming of HIV‐specific cellular responses, which may result in activation of HIV transcription within the expanded clonal T cell populations within this “vaccinal effect.” [43] However, a recent systematic review [4] reported that studies involving ATIs were not associated with the development of ART resistance, nor did the administration of immunotherapeutics, such as bNAbs, identify any safety concerns. The proportion of participants experiencing viral suppression by week 12 also reflect data from people living with HIV who are treatment‐naïve and starting integrase‐based ART for the first time [44, 45]. bNAbs were administered during ART‐mediated viral suppression in most of the included studies in this analysis, and were unlikely to explain the trend to a more rapid viral re‐suppression rate in the bNAb sensitivity analysis.

The strength of this meta‐analysis includes a robust systematic search strategy, completeness of the dataset to allow multivariable Cox proportional‐hazards analysis at an individual level and minimizing heterogeneity of the study data by tightening the search criteria to only studies after 2015, where ART regimen choices, in particular the use of integrase inhibitors, which have been demonstrated to result in more rapid viral suppression [44, 45], are likely to be similar across ATI studies. Furthermore, since 2019, recommendations from a consensus meeting on the conduct of ATI studies have been published [2], which may influence greater homogeneity across study designs involving ATIs in the future. Most ATI studies now recommend that participants who are on a non‐nucleoside reverse transcriptase inhibitors (NNRTIs)‐based ART regimen should be switched to either a protease inhibitor or integrase inhibitor, to avoid selection for NNRTI drug resistance due to their low barrier to resistance and long pharmacokinetic tail.

There are limitations to the generalizability of these results to address. Not all studies were randomized in this meta‐analysis, and adjusted differences seen between participants who received IA and those who underwent ATI alone may be due to unmeasured confounding variables not removed through a prospective randomization study design. The most common intervention therapeutic assessed within the meta‐analyses were bNAbs. Due to the small number of studies using other interventions available for inclusion in this systematic review, we were unable to rule out the individual effect of other non‐bNAb classes of therapeutics on time to viral suppression.

There was a lack of data on ART adherence, duration of ART use prior to ATI, and history of ART drug resistance at baseline or at the time of rebound were not available from some studies, thus we were unable to assess the contribution of these confounding variables to the primary outcome. The finding that a longer interval between HIV VL monitoring between ART restart and viral suppression was associated with longer times to viral suppression may represent a methodological bias where participants who suppress earlier after restarting ART may not be measured until their next VL measurement. Due to the limited availability of datapoints, only time to first undetectable VL was considered as the primary outcome, participants with persistent low‐level viraemia may be missed. At least one person (807) continued to experience low‐level viraemia throughout the study follow‐up period more than 2 years after restarting ART. The duration between HIV diagnosis and ART initiation was not consistently available across studies, and this may have also impacted on the size and diversity of the reservoir and subsequent time to viral re‐suppression in ATI studies.

We suggest future ATI studies should consider frequently monitoring plasma VL, at least monthly, until participants achieve undetectable readings after restarting ART. The development and implementation of validated point‐of‐care VL tests for participants to use at home or in the workplace may ease the burden of repeated clinic visits during these trials. Reporting the rates of sustained viral suppression (which may be defined as two or more undetectable readings after restarting ART, already in used in some studies [20]) for subsequent analyses, and routine genotyping of HIV sequences for ART‐resistance mutations at the time of VL rebound or ART restart if resources allows, should be considered in study protocols involving ATIs. Throughout the ATI studies and subsequent time to viral re‐suppression after restarting ART, participants and their partners should be offered access to HIV prevention strategies such as HIV pre‐exposure prophylaxis and condoms use to mitigate the risks of onward transmission. We would be cautious in extrapolating our findings to people who experience unstructured treatment interruptions, as this is a very different population with distinct characteristics, facing different barriers to adherence, ART regimens and HIV characteristics, and thus would be outside the scope of this paper.

## 5 CONCLUSIONS

In ATI studies included in this systematic review, the majority of participants achieved viral suppression to < 50 copies RNA/ml within 12 weeks of restarting ART. The receipt of interventional immune‐modulatory treatments appeared to be associated with a longer time to re‐suppression after adjustment for covariables compared to participants who underwent ATI alone. Integrase inhibitors were associated with more rapid re‐suppression rates and appeared to reverse the differences observed in time to viral re‐suppression. When designing studies involving ATIs, ART regimens containing integrase inhibitors should be considered on completion of ATIs, and the time to viral re‐suppression after restarting ART should be frequently monitored and reported, to ensure the safety of specific trial interventions in ATI studies.

## COMPETING INTERESTS

MJL is supported by the UK Medical Research Council Clinical Research Training Fellowship (MR/W024454/1), and has received speakers’ fees, conference from Viiv Healthcare, Gilead Sciences, and consultancy fees from Thriva Limited, external to the submitted work. EF has received grants and support for attending meetings from Viiv Healthcare and Gilead. BM has received stock options and consulting fees from AELIX Therapeutics SL, consulting fees from AbbviE, and speakers’ fees from Gilead, Janssen and Viiv Healthcare, outside the submitted work; and also participates on the data safety monitoring board for Leyden Labs. JLR is funded by the following NIH grants (NIH U19AI117950, NIH UM1AI164570) and has received stock options, royalties or licenses from Kite Pharma, BlueWhale Bio, and consulting fees from Scietemex Consulting, all external to the submitted work. JDG is supported by the Lundbeck Foundation (R381–2021–1405). All other authors have declared no conflicts of interests.

## AUTHORS’ CONTRIBUTIONS

MJL, ME and SF conceived the study. MJL and ME did the literature search, data extraction, statistical analysis and drafted the primary draft of the manuscript. AC, LG, MADS, JLR, PT, JDG, OSS, EF, EK, MDS, BM and MC contributed data from individual studies included in the analyses. MJL, ME and SF wrote the initial manuscript draft. All authors contributed critically to the manuscript and revisions, and approved the final version of the manuscript.

## FUNDING

This work is supported by the UK Medical Research Council, Imperial College NIHR BRC, MR/W024454/1..

## Supporting information


**Figure S1**. Sensitivity analysis: Kaplan Meier for people in bNAbs + ATI vs ATI only
**Figure S2**. Funnel plot of included studies
**Table S1**. Prisma checklist (separate file)
**Table S2**. Search strategies for systematic review on the Medline database till 22 Apr 2024
**Table S3**. Search strategies for systematic review on the Embase database till 22 Apr 2024
**Table S4**. Multivariable analysis for people in bNAbs + ATI only vs ATI only
**Table S5**. Search strategies for systematic review on the Web of Science database till 22 Apr 2024

PRISMA 2020 Checklist

## Data Availability

The template data collection form, data extracted from included studies and used for analyses, analytic code, and other material used in the review are available upon reasonable request to the corresponding author.
