# Supplementary material for: The impact of analytical treatment interruptions and trial interventions on time to viral re‐suppression in people living with HIV restarting ART in cure‐related clinical studies: a systematic review and meta‐analysis
Source: J Int AIDS Soc. 2024 Aug 18;27(8):e26349. doi: 10.1002/jia2.26349 (PMC11330850; doi:10.1002/jia2.26349)
Supplement: Supplementary file 1 — Figure S1. Sensitivity analysis: Kaplan Meier for people in bNAbs + ATI vs ATI only Figure S2. Funnel plot of included studies Table S1. Prisma checklist (separate file) Table S2. Search strategies for systematic review on the Medline database till 22 Apr 2024 Table S3. Search strategies for systematic review on the Embase database till 22 Apr 2024 Table S4. Multivariable analysis for people in bNAbs + ATI only vs ATI only Table S5. Search strategies for systematic review on the Web of Science database till 22 Apr 2024 [file JIA2-27-e26349-s001.docx]

**Supplementary material**

Supplementary Figure 1. Sensitivity analysis: Kaplan Meier for people in bNAbs + ATI vs ATI only


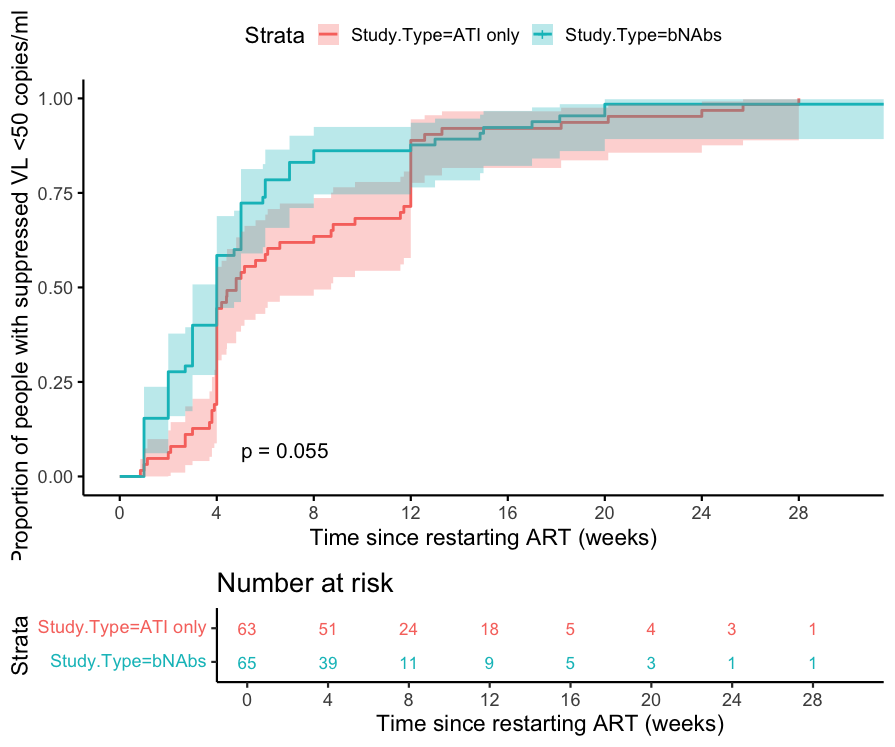


Supplementary Figure 1 legend. Kaplan Meier survival plot of proportion of people with suppressed plasma viral load <50 copies/ml against time in weeks. The red line represents participants undergoing ATI only protocols, and blue line represent participants who received bNAbs with ATI as part of their study protocol. The red and blue shaded areas represent the 95% confidence intervals of their respective lines. The table below shoes the numbers at risk at 4-weekly intervals stratified by receipt of interventional study drug with ATI or ATI-only protocols. Datapoints are censored past week 32. Abbreviations : ART – antiretroviral therapy; ATI – analytical treatment interruption; bNAbs – broadly neutralising antibodies; VL - viral load


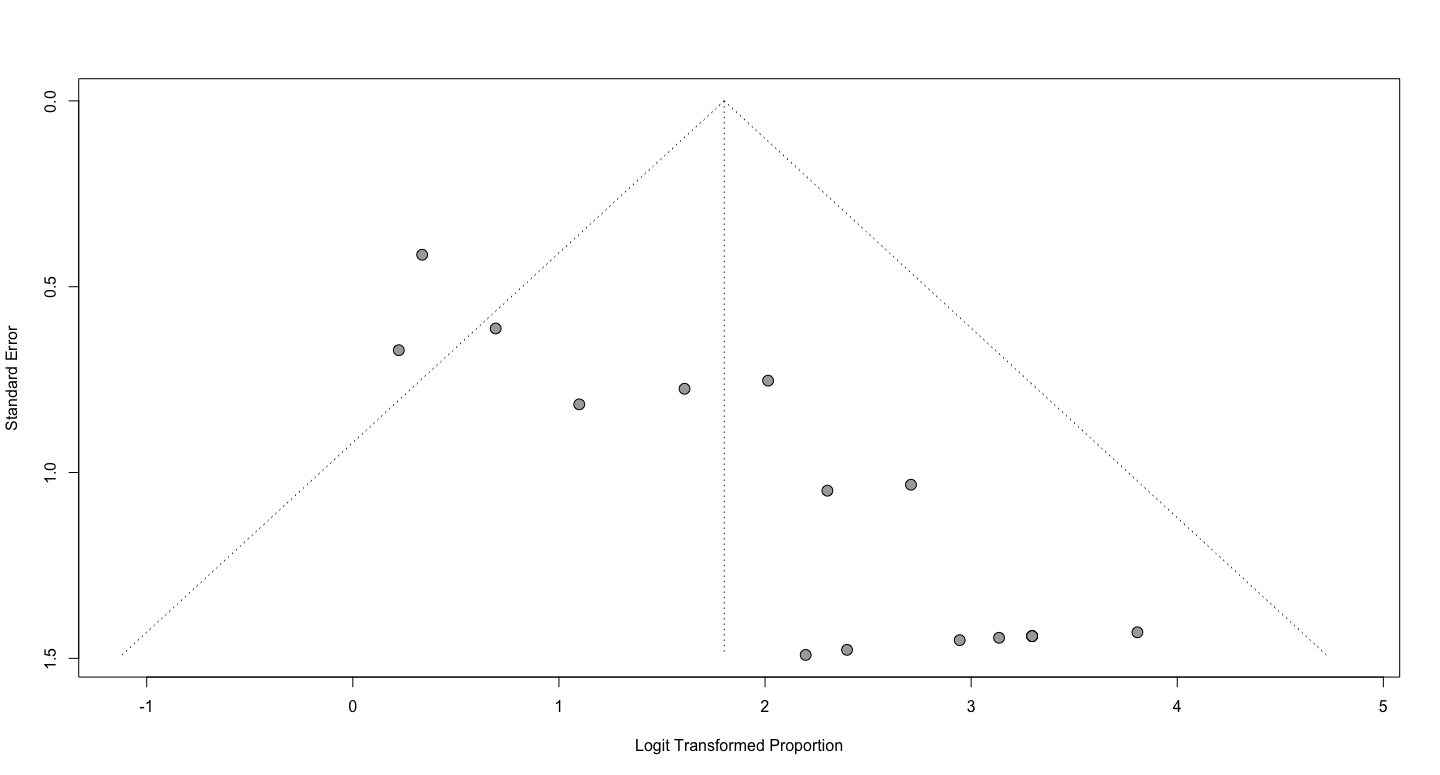
Supplementary Figure 2. Funnel plot of included studies

Supplementary table 1. Prisma checklist (separate file)

Supplementary table 2. Search strategies for systematic review on the Medline database till 22 Apr 2024

| **#** | **Query** | **Results from Medline** |
| --- | --- | --- |
| **1** | exp HIV/ or exp HIV-1/ | 109142 |
| **2** | exp Antiretroviral Therapy, Highly Active/ or exp Anti-Retroviral Agents/ or exp Anti-HIV Agents/ | 101243 |
| **3** | Pause*.mp. | 12724 |
| **4** | Stop*.mp. | 165587 |
| **5** | Discontinu*.mp. | 159640 |
| **6** | Interrupt*.mp | 90263 |
| **7** | exp Therapeutics/ | 5327024 |
| **8** | Drug*.mp. | 6811851 |
| **9** | ART.mp. | 175030 |
| **10** | 2 or 7 or 8 or 9 | 10535853 |
| **11** | 3 or 4 or 5 or 6 | 414160 |
| **12** | 1 and 10 and 11 | 2988 |
| **13** | limit 12 to (english language and humans and yr="2015 -Current" and "all adult (18 plus years)" and english and last 9 years) | 430 |

Supplementary table 3. Search strategies for systematic review on the Embase database till 22 Apr 2024

| **#** | **Query** | **Results from Embase** |
| --- | --- | --- |
| 1 | aids virus.mp. [mp=title, abstract, heading word, drug trade name, original title, device manufacturer, drug manufacturer, device trade name, keyword heading word, floating subheading word, candidate term word] | 1,088 |
| 2 | aids viruses.mp. [mp=title, abstract, heading word, drug trade name, original title, device manufacturer, drug manufacturer, device trade name, keyword heading word, floating subheading word, candidate term word] | 93 |
| 3 | acquired immune deficiency syndrome virus.mp. [mp=title, abstract, heading word, drug trade name, original title, device manufacturer, drug manufacturer, device trade name, keyword heading word, floating subheading word, candidate term word] | 18 |
| 4 | acquired immunodeficiency syndrome virus.mp. [mp=title, abstract, heading word, drug trade name, original title, device manufacturer, drug manufacturer, device trade name, keyword heading word, floating subheading word, candidate term word] | 32 |
| 5 | hiv.mp. [mp=title, abstract, heading word, drug trade name, original title, device manufacturer, drug manufacturer, device trade name, keyword heading word, floating subheading word, candidate term word] | 481070 |
| 6 | htlv-iii.mp. [mp=title, abstract, heading word, drug trade name, original title, device manufacturer, drug manufacturer, device trade name, keyword heading word, floating subheading word, candidate term word] | 1869 |
| 7 | human immunodeficiency virus.mp. [mp=title, abstract, heading word, drug trade name, original title, device manufacturer, drug manufacturer, device trade name, keyword heading word, floating subheading word, candidate term word] | 524380 |
| 8 | human immunodeficiency viruses.mp. [mp=title, abstract, heading word, drug trade name, original title, device manufacturer, drug manufacturer, device trade name, keyword heading word, floating subheading word, candidate term word] | 893 |
| 9 | human t cell leukemia virus type iii.mp. [mp=title, abstract, heading word, drug trade name, original title, device manufacturer, drug manufacturer, device trade name, keyword heading word, floating subheading word, candidate term word] | 16 |
| 10 | human t cell lymphotropic virus type iii.mp. [mp=title, abstract, heading word, drug trade name, original title, device manufacturer, drug manufacturer, device trade name, keyword heading word, floating subheading word, candidate term word] | 177 |
| 11 | human t lymphotropic virus type iii.mp. [mp=title, abstract, heading word, drug trade name, original title, device manufacturer, drug manufacturer, device trade name, keyword heading word, floating subheading word, candidate term word] | 229 |
| 12 | immunodeficiency virus, human.mp. [mp=title, abstract, heading word, drug trade name, original title, device manufacturer, drug manufacturer, device trade name, keyword heading word, floating subheading word, candidate term word] | 107 |
| 13 | immunodeficiency viruses, human.mp. [mp=title, abstract, heading word, drug trade name, original title, device manufacturer, drug manufacturer, device trade name, keyword heading word, floating subheading word, candidate term word] | 4 |
| 14 | lav-htlv-iii.mp. [mp=title, abstract, heading word, drug trade name, original title, device manufacturer, drug manufacturer, device trade name, keyword heading word, floating subheading word, candidate term word] | 240 |
| 15 | lymphadenopathy associated virus.mp. [mp=title, abstract, heading word, drug trade name, original title, device manufacturer, drug manufacturer, device trade name, keyword heading word, floating subheading word, candidate term word] | 299 |
| 16 | lymphadenopathy-associated viruses.mp. [mp=title, abstract, heading word, drug trade name, original title, device manufacturer, drug manufacturer, device trade name, keyword heading word, floating subheading word, candidate term word] | 0 |
| 17 | virus, aids.mp. [mp=title, abstract, heading word, drug trade name, original title, device manufacturer, drug manufacturer, device trade name, keyword heading word, floating subheading word, candidate term word] | 231 |
| 18 | virus, human immunodeficiency.mp. [mp=title, abstract, heading word, drug trade name, original title, device manufacturer, drug manufacturer, device trade name, keyword heading word, floating subheading word, candidate term word] | 390 |
| 19 | virus, lymphadenopathy-associated.mp. [mp=title, abstract, heading word, drug trade name, original title, device manufacturer, drug manufacturer, device trade name, keyword heading word, floating subheading word, candidate term word] | 11 |
| 20 | viruses, aids.mp. [mp=title, abstract, heading word, drug trade name, original title, device manufacturer, drug manufacturer, device trade name, keyword heading word, floating subheading word, candidate term word] | 6 |
| 21 | viruses, human immunodeficiency.mp. [mp=title, abstract, heading word, drug trade name, original title, device manufacturer, drug manufacturer, device trade name, keyword heading word, floating subheading word, candidate term word] | 115 |
| 22 | viruses, lymphadenopathy-associated.mp. [mp=title, abstract, heading word, drug trade name, original title, device manufacturer, drug manufacturer, device trade name, keyword heading word, floating subheading word, candidate term word] | 1 |
| 23 | txid12721.mp. [mp=title, abstract, heading word, drug trade name, original title, device manufacturer, drug manufacturer, device trade name, keyword heading word, floating subheading word, candidate term word] | 0 |
| 24 | hiv-1.mp. [mp=title, abstract, heading word, drug trade name, original title, device manufacturer, drug manufacturer, device trade name, keyword heading word, floating subheading word, candidate term word] | 106883 |
| 25 | hiv-i.mp. [mp=title, abstract, heading word, drug trade name, original title, device manufacturer, drug manufacturer, device trade name, keyword heading word, floating subheading word, candidate term word] | 1100 |
| 26 | human immunodeficiency virus type 1.mp. [mp=title, abstract, heading word, drug trade name, original title, device manufacturer, drug manufacturer, device trade name, keyword heading word, floating subheading word, candidate term word] | 22676 |
| 27 | human immunodeficiency virus 1.mp. [mp=title, abstract, heading word, drug trade name, original title, device manufacturer, drug manufacturer, device trade name, keyword heading word, floating subheading word, candidate term word] | 101666 |
| 28 | immunodeficiency virus type 1, human.mp. [mp=title, abstract, heading word, drug trade name, original title, device manufacturer, drug manufacturer, device trade name, keyword heading word, floating subheading word, candidate term word] | 13 |
| 29 | txid11676.mp. [mp=title, abstract, heading word, drug trade name, original title, device manufacturer, drug manufacturer, device trade name, keyword heading word, floating subheading word, candidate term word] | 0 |
| 30 | 24 or 25 or 26 or 27 or 28 or 29 | 132663 |
| 31 | 1 or 2 or 3 or 4 or 5 or 6 or 7 or 8 or 9 or 10 or 11 or 12 or 13 or 14 or 15 or 16 or 17 or 18 or 19 or 20 or 21 or 22 or 23 | 591860 |
| 32 | antiretroviral therapies, combination.mp. [mp=title, abstract, heading word, drug trade name, original title, device manufacturer, drug manufacturer, device trade name, keyword heading word, floating subheading word, candidate term word] | 0 |
| 33 | antiretroviral therapy, combination.mp. [mp=title, abstract, heading word, drug trade name, original title, device manufacturer, drug manufacturer, device trade name, keyword heading word, floating subheading word, candidate term word] | 16 |
| 34 | antiretroviral therapy, highly active.mp. [mp=title, abstract, heading word, drug trade name, original title, device manufacturer, drug manufacturer, device trade name, keyword heading word, floating subheading word, candidate term word] | 101 |
| 35 | combination antiretroviral therapies.mp. [mp=title, abstract, heading word, drug trade name, original title, device manufacturer, drug manufacturer, device trade name, keyword heading word, floating subheading word, candidate term word] | 137 |
| 36 | combination antiretroviral therapy.mp. [mp=title, abstract, heading word, drug trade name, original title, device manufacturer, drug manufacturer, device trade name, keyword heading word, floating subheading word, candidate term word] | 5353 |
| 37 | haart.mp. [mp=title, abstract, heading word, drug trade name, original title, device manufacturer, drug manufacturer, device trade name, keyword heading word, floating subheading word, candidate term word] | 19122 |
| 38 | therapies, combination antiretroviral.mp. [mp=title, abstract, heading word, drug trade name, original title, device manufacturer, drug manufacturer, device trade name, keyword heading word, floating subheading word, candidate term word] | 0 |
| 39 | therapy, combination antiretroviral.mp. [mp=title, abstract, heading word, drug trade name, original title, device manufacturer, drug manufacturer, device trade name, keyword heading word, floating subheading word, candidate term word] | 4 |
| 40 | 32 or 33 or 34 or 35 or 36 or 37 or 38 or 39 | 24474 |
| 41 | agent, antiretroviral.mp. [mp=title, abstract, heading word, drug trade name, original title, device manufacturer, drug manufacturer, device trade name, keyword heading word, floating subheading word, candidate term word] | 9 |
| 42 | agents, anti-retroviral.mp. [mp=title, abstract, heading word, drug trade name, original title, device manufacturer, drug manufacturer, device trade name, keyword heading word, floating subheading word, candidate term word] | 2 |
| 43 | anti retroviral agents.mp. [mp=title, abstract, heading word, drug trade name, original title, device manufacturer, drug manufacturer, device trade name, keyword heading word, floating subheading word, candidate term word] | 230 |
| 44 | antiretroviral agent.mp. [mp=title, abstract, heading word, drug trade name, original title, device manufacturer, drug manufacturer, device trade name, keyword heading word, floating subheading word, candidate term word] | 453 |
| 45 | 41 or 42 or 43 or 44 | 691 |
| 46 | aids drug.mp. [mp=title, abstract, heading word, drug trade name, original title, device manufacturer, drug manufacturer, device trade name, keyword heading word, floating subheading word, candidate term word] | 682 |
| 47 | aids drugs.mp. [mp=title, abstract, heading word, drug trade name, original title, device manufacturer, drug manufacturer, device trade name, keyword heading word, floating subheading word, candidate term word] | 455 |
| 48 | agent, anti-hiv.mp. [mp=title, abstract, heading word, drug trade name, original title, device manufacturer, drug manufacturer, device trade name, keyword heading word, floating subheading word, candidate term word] | 2 |
| 49 | agents, anti-aids.mp. [mp=title, abstract, heading word, drug trade name, original title, device manufacturer, drug manufacturer, device trade name, keyword heading word, floating subheading word, candidate term word] | 1 |
| 50 | agents, anti-hiv.mp. [mp=title, abstract, heading word, drug trade name, original title, device manufacturer, drug manufacturer, device trade name, keyword heading word, floating subheading word, candidate term word] | 13 |
| 51 | anti aids agents.mp. [mp=title, abstract, heading word, drug trade name, original title, device manufacturer, drug manufacturer, device trade name, keyword heading word, floating subheading word, candidate term word] | 107 |
| 52 | anti aids drug.mp. [mp=title, abstract, heading word, drug trade name, original title, device manufacturer, drug manufacturer, device trade name, keyword heading word, floating subheading word, candidate term word] | 124 |
| 53 | anti aids drugs.mp. [mp=title, abstract, heading word, drug trade name, original title, device manufacturer, drug manufacturer, device trade name, keyword heading word, floating subheading word, candidate term word] | 127 |
| 54 | anti hiv agent.mp. [mp=title, abstract, heading word, drug trade name, original title, device manufacturer, drug manufacturer, device trade name, keyword heading word, floating subheading word, candidate term word] | 450 |
| 55 | anti hiv agents.mp. [mp=title, abstract, heading word, drug trade name, original title, device manufacturer, drug manufacturer, device trade name, keyword heading word, floating subheading word, candidate term word] | 1,326 |
| 56 | anti hiv drug.mp. [mp=title, abstract, heading word, drug trade name, original title, device manufacturer, drug manufacturer, device trade name, keyword heading word, floating subheading word, candidate term word] | 786 |
| 57 | anti hiv drugs.mp. [mp=title, abstract, heading word, drug trade name, original title, device manufacturer, drug manufacturer, device trade name, keyword heading word, floating subheading word, candidate term word] | 1,338 |
| 58 | drug, aids.mp. [mp=title, abstract, heading word, drug trade name, original title, device manufacturer, drug manufacturer, device trade name, keyword heading word, floating subheading word, candidate term word] | 13 |
| 59 | drug, anti-aids.mp. [mp=title, abstract, heading word, drug trade name, original title, device manufacturer, drug manufacturer, device trade name, keyword heading word, floating subheading word, candidate term word] | 1 |
| 60 | drug, anti-hiv.mp. [mp=title, abstract, heading word, drug trade name, original title, device manufacturer, drug manufacturer, device trade name, keyword heading word, floating subheading word, candidate term word] | 9 |
| 61 | drugs, aids.mp. [mp=title, abstract, heading word, drug trade name, original title, device manufacturer, drug manufacturer, device trade name, keyword heading word, floating subheading word, candidate term word] | 42 |
| 62 | drugs, anti-aids.mp. [mp=title, abstract, heading word, drug trade name, original title, device manufacturer, drug manufacturer, device trade name, keyword heading word, floating subheading word, candidate term word] | 0 |
| 63 | drugs, anti-hiv.mp. [mp=title, abstract, heading word, drug trade name, original title, device manufacturer, drug manufacturer, device trade name, keyword heading word, floating subheading word, candidate term word] | 8 |
| 64 | 46 or 47 or 48 or 49 or 50 or 51 or 52 or 53 or 54 or 55 or 56 or 57 or 58 or 59 or 60 or 61 or 62 or 63 | 4840 |
| 65 | pause*.mp. [mp=title, abstract, heading word, drug trade name, original title, device manufacturer, drug manufacturer, device trade name, keyword heading word, floating subheading word, candidate term word] | 19131 |
| 66 | Stop*.mp. [mp=title, abstract, heading word, drug trade name, original title, device manufacturer, drug manufacturer, device trade name, keyword heading word, floating subheading word, candidate term word] | 276296 |
| 67 | Discontinu*.mp. [mp=title, abstract, heading word, drug trade name, original title, device manufacturer, drug manufacturer, device trade name, keyword heading word, floating subheading word, candidate term word] | 283679 |
| 68 | Interrupt*.mp. | 137726 |
| 69 | 65 or 66 or 67 or 68 | 684111 |
| 70 | (Treatment or Treatments or therapeutic or therapeutics or therapy or therapies).mp. [mp=title, abstract, heading word, drug trade name, original title, device manufacturer, drug manufacturer, device trade name, keyword heading word, floating subheading word, candidate term word] | 15379826 |
| 71 | ART.mp. [mp=title, abstract, heading word, drug trade name, original title, device manufacturer, drug manufacturer, device trade name, keyword heading word, floating subheading word, candidate term word] | 265677 |
| 72 | drug*.mp. [mp=title, abstract, heading word, drug trade name, original title, device manufacturer, drug manufacturer, device trade name, keyword heading word, floating subheading word, candidate term word] | 14359545 |
| 73 | 40 or 45 or 64 or 70 or 71 or 72 | 21493210 |
| 74 | 30 and 31 and 69 and 73 | 4608 |
| 75 | limit 74 to (human and english language and embase and english and yr="2015 -Current" and adult <18 to 99 years> and last 9 years) | 578 |

Supplementary table 5. Search strategies for systematic review on the Web of Science database till 22 Apr 2024

| **#** | **Query** | **Results from Wos** |
| --- | --- | --- |
| **1** | **HIV** (All Fields) or **HIV-1** (All Fields) | 466970 |
| **2** | **TS=("Antiretroviral Therapy, Highly Active" OR "Antiretroviral Agents" OR "Anti-HIV agents" OR "Therapeutics" OR "Drug" OR "ART")** | 2738328 |
| **3** | **TS=("Pause" OR "Stop" OR "Discontinue" OR "Interrupt")** | 171595 |
| **4** | **#1 AND #2 AND #3** | 786 |
| **5** | **#4 AND English (Languages) AND 2015 TO 2024 (Publication Year)** | 407 |

Supplementary table 4. Multivariable analysis for people in bNAbs + ATI only vs ATI only

|  |  | N (%) /  Mean (SD) | Univariable | | | Multivariable | | |
| --- | --- | --- | --- | --- | --- | --- | --- | --- |
|  |  |  | HR | 95% CI | p-value | HR | 95% CI | p-value |
| **Patient characteristics** |  |  |  |  |  |  |  |  |
| Age (years) (per 10-year increase) |  | 42.4 (10.9) | 0.79 | 0.67 – 0.94 | 0.007 | 0.89 | 0.72 – 1.11 | 0.295 |
| Sex^+^ | Female (Ref) | 10 (8.8) |  |  |  |  |  |  |
|  | Male | 104 (91.2) | 0.73 | 0.38 – 1.41 | 0.346 |  |  |  |
| **HIV-related characteristics** |  |  |  |  |  |  |  |  |
| Plasma HIV viral load at ART restart  (Log copies/ml) | | 4.3 (0.9) | 0.55 | 0.45 – 0.68 | <0.001 | 0.52 | 0.42 – 0.66 | **<0.001** |
| Nadir CD4 (per 100 cells/μl increase) | | 490.3 (277.7) | 1.00 | 0.93 - 1.07 | 0.745 |  |  |  |
| CD4 at study enrolment (per 100 cells/μl increase) | | 803.8 (310.0) | 0.92 | 0.87 – 0.98 | 0.007 | 0.96 | 0.89 – 1.02 | 0.197 |
| Duration since HIV diagnosis (years) | | 8.5 (7.6) | 0.98 | 0.96 – 1.01 | 0.120 | 0.97 | 0.93 – 1.00 | 0.057 |
| ART regimen containing Integrase Inhibitors | No (Ref) | 31 (24.4%) |  |  |  |  |  |  |
|  | Yes | 97 (75.6%) | 1.31 | 0.87 – 1.99 | 0.197 | 2.12 | 1.25 – 3.59 | **0.005** |
| **Study characteristics** |  |  |  |  |  |  |  |  |
| Duration of ATI in weeks |  | 9.7 (8.2) | 1.00 | 0.98 – 1.02 | 0.799 |  |  |  |
| Mean interval of HIV VL monitoring between ART restart and viral suppression (weeks) | | 3.3 (2.0) | 0.71 | 0.64 – 0.80 | <0.001 | 0.49 | 0.40 – 0.61 | **<0.001** |
| Study type* | ATI only (Ref) | 63 (49.6) |  |  |  |  |  |  |
|  | bNAbs and ATI | 65 (50.4) | 1.37 | 1.08 – 2.21 | 0.075 | 0.27 | 0.15 – 0.49 | **<0.001** |

+ A transgender participant was not included in the multivariable analysis due to n <5.

Abbreviations: Ref – Reference category; ART – antiretroviral therapy; ATI – analytical treatment interruption; IA – intervention with ATI protocol; HR – hazard ratio.
